# Supplementary material for: Electromagnon dispersion probed by inelastic X-ray scattering in LiCrO2
Source: Nat Commun. 2016 Nov 24;7:13547. doi: 10.1038/ncomms13547 (PMC5123047; doi:10.1038/ncomms13547)
Supplement: Supplementary Information — Supplementary Figures 1-4, Supplementary Table 1-4, Supplementary Note 1 and Supplementary References. [file ncomms13547-s1.pdf]

SUPPLEMENTARY FIGURES

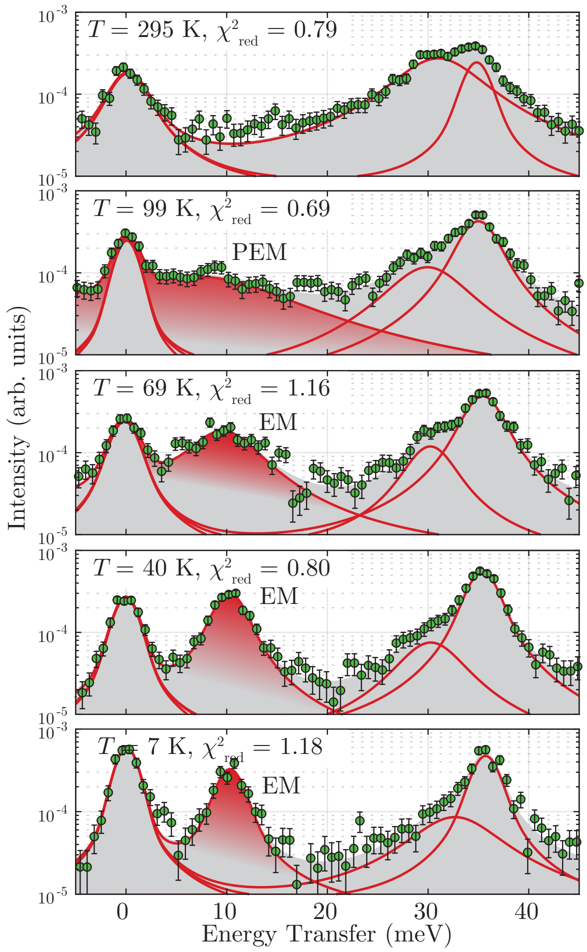

SUPPLEMENTARY FIGURE 1. **IXS data of $LiCrO_2$ measured at different temperatures.** Data was collected at $Q = (1.5, 1.5, 0)$ reciprocal space point. Intensity is shown in logarithmic scale and all datasets are normalised to the same monitor. Red lines show individual fitted peaks and grey shaded region shows the sum of the fitted peaks. Red shaded regions show the electromagnon (EM) and paraelectromagnon (PEM) peaks. The χ^2_{red} goodness of fit is given for each scan. Error bars indicate 1 s.d.

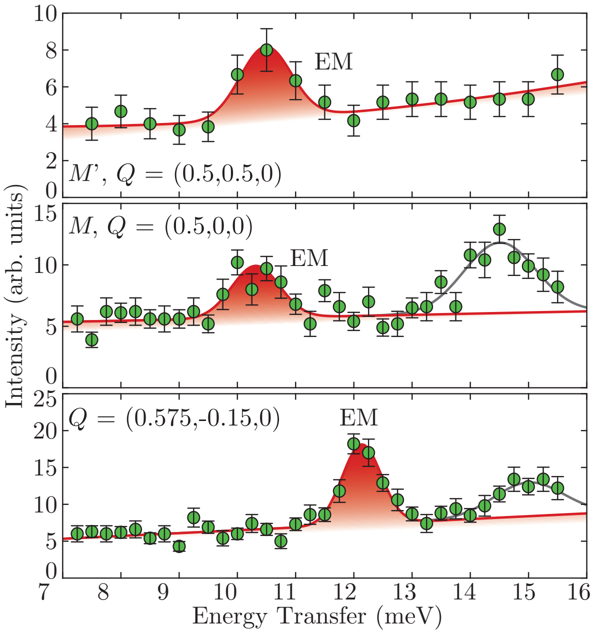

SUPPLEMENTARY FIGURE 2. **INS data of $LiCrO_2$.** The constant Q scans were measured at 1.5 K. The fitted red Gaussian peaks are the phason spin wave mode. Note that the grey peaks are instrumental artefacts. The fitted phason energies along $(h, 1-2h, 0)$ was found to be identical to the electromagnon dispersion measured with IXS along $(h, h, 0)$. Error bars indicate 1 s.d.

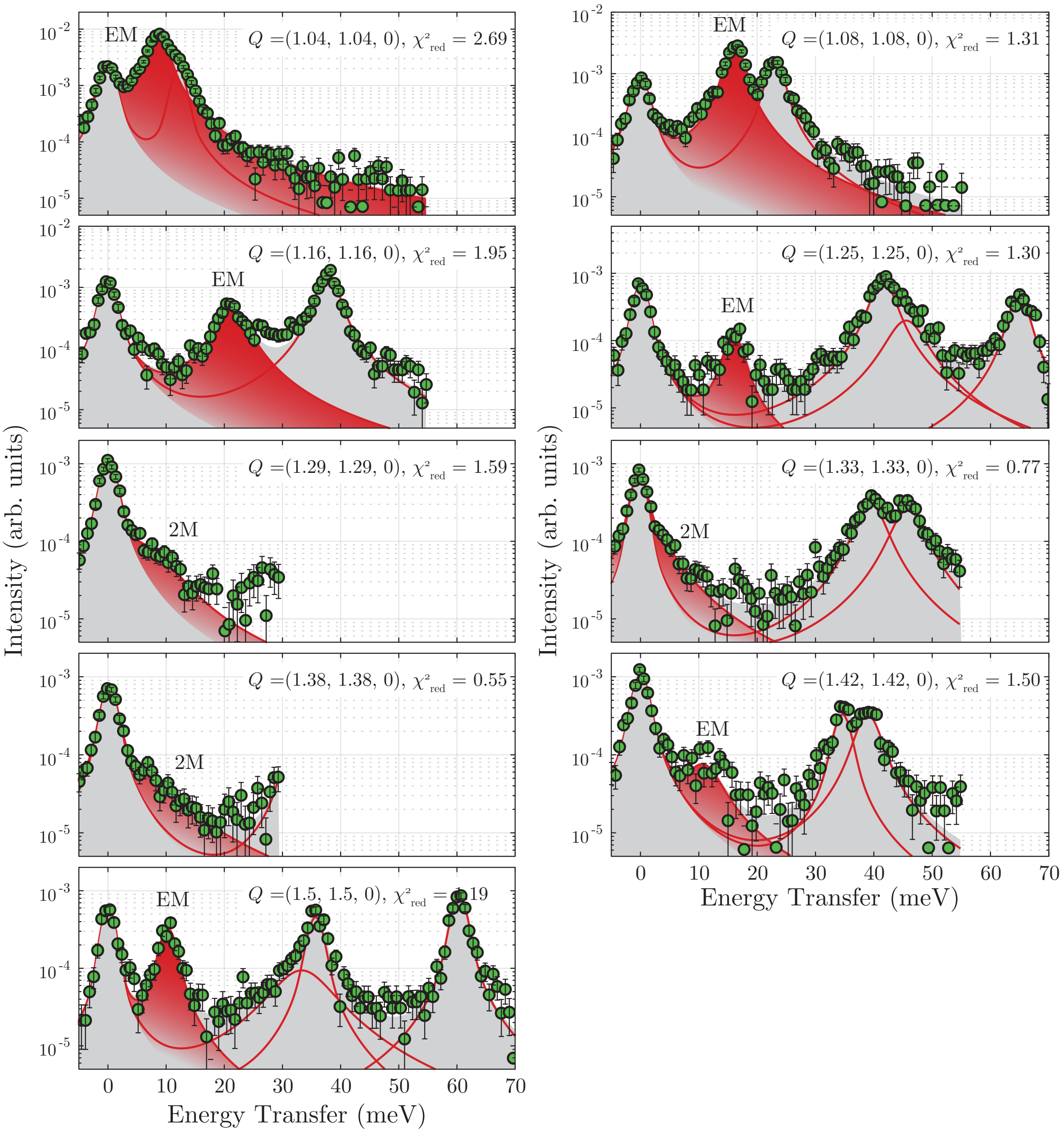

SUPPLEMENTARY FIGURE 3. **Low temperature IXS data of $LiCrO_2$.** The constant Q scans were measured along the $(h, h, 0)$ direction at 7 K. Intensity is shown in logarithmic scale and all datasets are normalised to the same monitor. Red lines show individual fitted peaks and grey shaded region shows the sum of the fitted peaks. Red shaded regions show the electromagnon and two magnon peaks. The χ^2_{red} reduced goodness of fit is given for each scan. Error bars indicate 1 s.d.

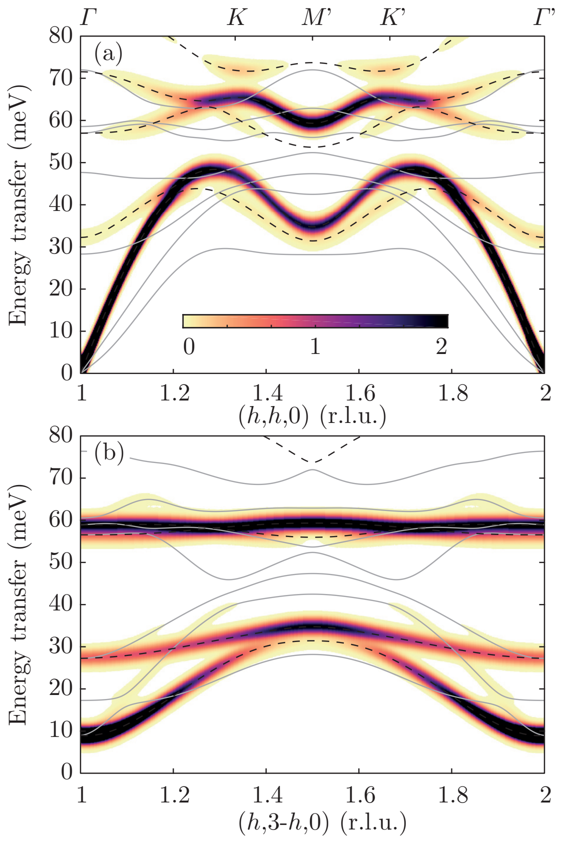

SUPPLEMENTARY FIGURE 4. **Pure phonon spectrum of $LiCrO_2$.** The phonon dynamical structure factor is calculated from *ab initio* methods. Dashed and grey lines denote longitudinal (with non-zero IXS cross section) and the transverse phonon modes (zero IXS cross section). The colormap shows the calculated IXS cross section in arbitrary units.

SUPPLEMENTARY TABLES

SUPPLEMENTARY TABLE 1. **Fitted electromagnon and phonon energies.** The peak positions are fitted to the IXS data measured at 7 K with error corresponding to 1 s.d.

$(h,h,0)$ (r.l.u.)	E_{EM} (meV)	E_{PH1} (meV)	E_{PH2} (meV)	E_{PH3} (meV)
1.0417	8.6(1)	12.1(2)		
1.0830	16.2(1)	23.1(1)		
1.1667	21.3(2)	37.8(1)		
1.2500	15.9(3)	41.5(3)	46(1)	64.8(2)
1.2916	7(1)			
1.3330	1.6(8)	39.7(3)	46.1(3)	
1.3750	5(2)	32(3)		
1.4167	10.8(9)	34.6(3)	39.0(4)	
1.5000	10.3(2)	33(1)	35.6(2)	60.3(1)

SUPPLEMENTARY TABLE 2. **Fitted electromagnon and phonon dynamical susceptibility.** The dynamical susceptibilities are fitted to the IXS data measured at 7 K with error corresponding to 1 s.d. All intensity is scaled up by a factor of 10^3 and are equal to the dynamical susceptibility up to a global scaling factor.

$(h,h,0)$ (r.l.u.)	I_{EM}	I_{PH1}	I_{PH2}	I_{PH3}
1.0417	41(2)	6(2)		
1.0830	13.6(4)	6.9(3)		
1.1667	3.8(2)	9.0(3)		
1.2500	0.59(8)	5(1)	2(1)	2.3(2)
1.2916	0.8(2)			
1.3330	1.3(3)	2.5(3)	2.3(3)	
1.3750	0.9(3)	1(5)		
1.4167	0.6(1)	1.5(4)	2.2(4)	
1.5000	1.5(1)	1.6(6)	1.9(6)	3.6(2)

SUPPLEMENTARY TABLE 3. **Fitted intrinsic peak widths.** All widths are fitted to the IXS data measured at 7 K with error corresponding to 1 s.d.

$(h, h, 0)$ (r.l.u.)	w_{EM} (meV)	w_{PH1} (meV)	w_{PH2} (meV)	w_{PH3} (meV)
1.0417	0.8(2)	-0.4(5)		
1.0830	0.7(1)	0.5(2)		
1.1667	3.1(5)	1.1(2)		
1.2500	0.9(7)	1.8(6)	4.3(16)	0.9(3)
1.2916	7(2)			
1.3330	6(2)	3.0(6)	3.6(8)	
1.3750	14(3)	1(10)		
1.4167	4(2)	0.5(7)	2.1(7)	
1.5000	0.5(4)	9(3)	0.2(7)	0.1(2)

SUPPLEMENTARY TABLE 4. **Bond vectors of the triangular lattice.** The xyz Descartes coordinate system is fixed to the lattice $x \| a$, y is in the ab-plane and z is perpendicular to the xy plane.

bond index	$\mathbf{d}_i$ in l.u.	$\mathbf{d}_i$ in xyz
1	(1,0,0)	$(1, 0, 0)$
2	(0,1,0)	$(-1/2, \sqrt{3}/2, 0)$
3	(1,1,0)	$(1/2, \sqrt{3}/2, 0)$
4	(-1,0,0)	$(-1, 0, 0)$
5	(0,-1,0)	$(1/2, -\sqrt{3}/2, 0)$
6	(-1,-1,0)	$(-1/2, -\sqrt{3}/2, 0)$

SUPPLEMENTARY NOTE

SUPPLEMENTARY NOTE 1. **Theory of magnon-phonon coupling on the triangular lattice.**

In this Supplementary Note we describe the calculation of the correlation function of the 2D Heisenberg triangular lattice antiferromagnet (TLA) with lattice and spins coupled via exchange striction. Similar calculation was published earlier by J. H. Kim and J. H. Han [1], however here we generalise the formalism to allow for arbitrary phonon dispersion and further neighbour couplings and incommensurate ordering wave vectors. Our method can be also easily generalised for non-Bravais lattices.

Coupled magnon-phonon model on the triangular lattice. In the following we will assume that exchange striction is the mechanism that couples the phonons to the magnons of an ordered magnetic ground state. The coupled Hamiltonian can be written as:

$$\mathcal{H} = \sum_{m,n} \left(J_1 - J_{mp} \hat{\mathbf{d}}_{mn} \cdot (\mathbf{u}_n - \mathbf{u}_m) \right) \mathbf{S}_m^{\intercal} \mathsf{R}_{mn} \mathbf{S}_n + \mathcal{H}_{\mathrm{ph}}, \tag{1}$$

where $J_1 > 0$ is the first neighbour antiferromagnetic exchange between spins, R_{mn} is a rotation matrix between cell m and n, that transforms the spiral magnetic ground state into a ferromagnet [2], $\mathbf{S}_m$ is the spin vector operator (column vector) and J_{mp} is the magnon-phonon coupling constant the derivative of J_1 as a function of bond length. This formalism applies to any Bravais lattice with single-k magnetic order.

The spin vector operators can be expressed with the magnon creation and annihilation operators after the linear Holstein-Primakoff approximation in the following form [2]:

$$\mathbf{S}_m = \sqrt{\frac{S}{2}} \left(\overline{\mathbf{u}} b_m + \mathbf{u} b_m^{\dagger} \right) + \mathbf{v} \left(S - b_m^{\dagger} b_m \right), \tag{2}$$

where $\mathbf{v}$ is a unit vector parallel to the spin direction (quantisation axis) and $\mathbf{u}$ is a complex vector where the real and imaginary part spanning the plane perpendicular to $\mathbf{v}$. For example the following vectors describe an ac-plane spin spiral:

$$\begin{aligned} \mathbf{v} &= (1, 0, 0), \\ \mathbf{u} &= (0, 1, i), \end{aligned} \tag{3}$$

with the corresponding rotation matrix:

$$\mathsf{R}_{mn} = \begin{pmatrix} \cos(2\pi\mathbf{Q}\mathbf{d}_{mn}) & 0 & -\sin(2\pi\mathbf{Q}\mathbf{d}_{mn}) \\ 0 & 1 & 0 \\ \sin(2\pi\mathbf{Q}\mathbf{d}_{mn}) & 0 & \cos(2\pi\mathbf{Q}\mathbf{d}_{mn}) \end{pmatrix}, \tag{4}$$

where $\mathbf{Q}$ is the magnetic ordering wave vector. Although in the main article we used $\mathbf{k}_m$ for the magnetic ordering wave vector here will will use $\mathbf{Q}$ for the same quantity to avoid confusion with summation index m.

To calculate the coupled magnon phonon spectrum we keep only the quadratic terms of the bosonic Hamiltonian. It can be shown using mean field theory that the constant and one operator terms does not change the crystal structure.[3] We also neglect higher order terms that are generally small for $S > 1$. The Hilbert space of the coupled model is the direct product of the magnetic and phononic Hilbert spaces. The quadratic coupled Hamiltonian in second quantised formalism is the direct sum of the quadratic matrices of the spin and phonon subsystem plus the coupling term appears in the off-diagonal blocks. It is important to note, that the linear coupling between phonons and magnons only happens if the magnetic order is non-collinear since in collinear magnets the one magnon term in the spin Hamiltonian is zero. In the following we work out the mixed magnon-phonon quadratic term and use the previously published quadratic magnon term [2].

The mixed magnon-phonon quadratic term is the following:

$$\begin{aligned} \mathcal{H}_{\text{mp}} &= J_{mp} \sum_{mn} \hat{\mathbf{d}}_{mn} \cdot (\mathbf{u}_n - \mathbf{u}_m) \mathcal{S}_{mn}, \\ \mathcal{S}_{mn} &= \sqrt{\frac{S^3}{2}} \{ (\overline{\mathbf{u}} b_m + \overline{\mathbf{u}} b_m^\dagger)^\intercal \mathsf{R}_{mn} \mathbf{v} + \mathbf{v} \mathsf{R}_{mn} (\overline{\mathbf{u}} b_n + \mathbf{u} b_n^\dagger) \}, \end{aligned} \tag{5}$$

where $\mathcal{S}_{mn}$ is the one operator term from the spin Hamiltonian which can be simplified to:

$$\mathcal{S}_{mn} = i\sqrt{\frac{S^3}{2}} \sin(2\pi\mathbf{Q}\mathbf{d}_{mn})(b_m^\dagger - b_m + b_n - b_n^\dagger). \tag{6}$$

This operator changes sign with time inversion and if coupled to a phonon operator invariant under time reversal, $\mathcal{H}_{\text{mp}}$ seemingly breaks time inversion symmetry. However our Hamiltonian is linearised on the assumption of a time reversal breaking ground state of a spin spiral. It is also easy to see from Supplementary Equation 6, that if $2\mathbf{Q}$ is equal to a lattice vector (collinear magnetic structures on the Bravais lattice) the magnon-phonon mixing term is zero.

To diagonalise the quadratic Hamiltonian, we Fourier transform the b_m and $b_m^\dagger$ operators using the formula:

$$b_m = \frac{1}{\sqrt{L}} \sum_{\mathbf{k} \in B.Z.} b(\mathbf{k}) e^{2\pi i \mathbf{k}\cdot\mathbf{r}_m}, \tag{7}$$

where L is the number of atoms in the system and the summation runs over the Brillouin zone. After the Fourier transformation and some algebra we arrive at the following formula for the mixed magnon-phonon term on the Bravais lattice:

$$\mathcal{H}_{\mathrm{mp}} = i \sum_{\mathbf{k},\lambda} \gamma_\lambda(\mathbf{k}) a_\lambda(\mathbf{k}) \left(b^\dagger(\mathbf{k}) - b(-\mathbf{k})\right) + \mathrm{h.c.}, \tag{8}$$

where the coupling amplitude $\gamma_\lambda(\mathbf{k})$ is defined as:

$$\gamma_\lambda(\mathbf{k}) = -\frac{3}{4} J_{\mathrm{mp}} \sqrt{\frac{S^3 \hbar}{M \omega_\lambda(\mathbf{k})}} \mathbf{e}_\lambda(\mathbf{k}) \cdot \mathbf{g}(\mathbf{k}), \tag{9}$$

with the geometrical factor $\mathbf{g}(\mathbf{k})$ that depends on the lattice geometry:

$$\mathbf{g}(\mathbf{k}) = \sum_{\mathbf{d}} \sin(2\pi \mathbf{Q}\cdot\mathbf{d}) \left[\cos(2\pi \mathbf{k}\cdot\mathbf{d}) - 1\right] \hat{\mathbf{d}}. \tag{10}$$

The summation runs over the $\mathbf{d}$ bond vectors where exchange striction is active. To get an estimation for the size of the matrix elements, the phonon displacement can be calculated in absolute units: $u_0 = \sqrt{\hbar/M/\omega_\lambda(\mathbf{k})}$ for M set to the atomic mass units and $\omega_\lambda(\mathbf{k})$ to 1 meV, the phonon amplitude unit is then $u_0 = 5.125$ Å.

Now we develop the coupled equations for the triangular lattice antiferromagnet. We assume that the exchange striction is active on the first neighbour bonds within the triangular plane, while further neighbour bonds don't contribute to the magnon-phonon coupling. We also restrict the calculation to antiferromagnetic first neighbour exchange and the magnetic structure to be a $\mathbf{Q} = (1/3, 1/3, 0)$ spiral. For the evaluation of the $\mathbf{Q}\cdot\mathbf{d}$ expressions, the easiest is to use the lattice coordinate system, see Supplementary Table 4. For evaluation of the $\mathbf{d}\cdot\mathbf{e}_\lambda(\mathbf{k})$ expressions the best coordinate system is the Descartes coordinate system fixed to the lattice ($x \| a$, y is in the ab-plane). After doing the summation in Supplementary Equation 10 we get the vector components of the geometrical coupling factor in the xyz coordinate system:

$$\begin{aligned} \sqrt{3} g^x(\mathbf{k}) &= 2\cos(2\pi \mathbf{k}\mathbf{d}_1) - \cos(2\pi \mathbf{k}\mathbf{d}_2) - \cos(2\pi \mathbf{k}\mathbf{d}_3), \\ g^y(\mathbf{k}) &= \cos(2\pi \mathbf{k}\mathbf{d}_2) - \cos(2\pi \mathbf{k}\mathbf{d}_3). \end{aligned} \tag{11}$$

For the $\mathbf{k} = (h, h, 0)$ direction in reciprocal space, the geometrical coupling vector is:

$$\mathbf{g}(\mathbf{k}) = \mathbf{d}_3 f(\mathbf{k}). \tag{12}$$

which means the magnons couple only to longitudinal phonons while it can be shown that along the $\mathbf{k} = (0, k, 0)$ reciprocal space direction the magnons couple to phonons polarised along $(1, 0, 0)$.

Phonon dynamical correlation function. Inelastic x-ray scattering in non-resonant condition measures the atomic displacement correlation function:

$$S_{\mathrm{ph}}(\mathbf{k}, \omega) = \frac{1}{2\pi L^2} \sum_{m,i,n,j} e^{i2\pi \mathbf{k}(\mathbf{r}_{mi} - \mathbf{r}_{nj})} \int_{-\infty}^{\infty} d\tau e^{\imath\omega\tau} \langle \mathbf{u}_{mi} \mathbf{u}_{nj}^{\intercal}(\tau) \rangle. \tag{13}$$

The summation runs over the m and n unit cell indices in the crystal with L unit cells and the N atoms within the unit cell with index i and j.

The summation over the unit cells can be expressed as:

$$U_{ij}(\tau) = \sum_{m,n} e^{i\mathbf{k}(\mathbf{r}_{mi} - \mathbf{r}_{nj})} \langle \mathbf{u}_{mi} \mathbf{u}_{nj}^{\intercal}(\tau) \rangle = \sum_{\lambda\lambda'} \frac{\hbar \mathbf{e}_{\lambda}^{i*}(\mathbf{k}) \mathbf{e}_{\lambda'}^{j\intercal}(\mathbf{k})}{2\sqrt{\omega_\lambda(\mathbf{k}) \omega_{\lambda'}(\mathbf{k}) M_i M_j}} e^{i\mathbf{k}(\mathbf{t}_i - \mathbf{t}_j)}$$
$$\langle a_\lambda(-\mathbf{k}) a_{\lambda'}(\mathbf{k}, \tau) + a_\lambda^+(\mathbf{k}) a_{\lambda'}(\mathbf{k}, \tau) + a_\lambda(-\mathbf{k}) a_{\lambda'}^+(-\mathbf{k}, \tau) + a_\lambda^+(\mathbf{k}) a_{\lambda'}^+(-\mathbf{k}, \tau) \rangle \tag{14}$$

To simplify the calculation we introduce the matrix V with the ith row is defined as:

$$\mathbf{V}_i^\alpha = \sqrt{\frac{\hbar}{2M_i}} e^{-i\mathbf{k}\mathbf{t}_i} \frac{\mathbf{e}_i^\alpha(\mathbf{k})}{\sqrt{\omega(\mathbf{k})}}, \tag{15}$$

where α indexes the three xyz spatial components, $\mathbf{e}_i^\alpha$ is the N component vector of the α component of the phonon polarisation vectors, $\omega(\mathbf{k})$ is the vector of the $\omega_\lambda(\mathbf{k})$ phonon eigenvalues at a specific k vector. The size of V^α is $N \times 3N$. Using the column vector of both the magnon and phonon boson operators defined as:

$$\mathbf{x}(\mathbf{k}) = \left[a_1(\mathbf{k}) \ldots a_{3N}(\mathbf{k}) b(\mathbf{k}) a_1^+(-\mathbf{k}) \ldots a_{3N}^+(-\mathbf{k}) b^+(-\mathbf{k})\right]^\intercal, \tag{16}$$

the displacement matrix after summation over the atoms in the unit cell can be expressed as:

$$\sum_{ij} U_{ij}^{\alpha\beta} = \langle \mathbf{x}^{+}(\mathbf{k}) \begin{bmatrix} \mathbf{V}^{\alpha+}(\mathbf{k}) \\ 0 \\ \mathbf{V}^{\alpha+}(\mathbf{k}) \\ 0 \end{bmatrix} \otimes \left[\mathbf{V}^{\beta}(\mathbf{k}), 0, \mathbf{V}^{\beta}(\mathbf{k}), 0\right] \mathbf{x}(\mathbf{k},\tau)\rangle, \tag{17}$$

where

$$\mathbf{V}(\mathbf{k}) = \sum_i \mathbf{V}_i^{\alpha}(\mathbf{k}). \tag{18}$$

Using the unitary transformation T (omitting the explicit $\mathbf{k}$ dependence) that diagonalises the magnon-phonon Hamiltonian [2] the $\mathbf{x}'$ vector of the non-interacting boson operators can be expressed as:

$$\mathbf{x} = \mathsf{T}\mathbf{x}'. \tag{19}$$

The expectation value of the noon-interacting boson operators are:

$$\begin{aligned} \langle x_i'^{+}(\mathbf{k}), x_j'(\mathbf{k},\tau)\rangle &= \delta_{ij} n(\omega_i'(\mathbf{k})) e^{-i\omega_i'(\mathbf{k})\tau} \\ \langle x_i'(\mathbf{k}), x_j'^{+}(\mathbf{k},\tau)\rangle &= \delta_{ij} \left(1 + n(\omega_i'(\mathbf{k}))\right) e^{-i\omega_i'(\mathbf{k})\tau}, \end{aligned} \tag{20}$$

where $n(\omega)$ is the Bose-Einstein statistics of the bosons and $\omega_i'(\mathbf{k})$ are the eigenvalues of the coupled Hamiltonian, not to mix with the phonon eigenvalues.

Finally the displacement correlation function can be expressed as:

$$S_{ph}^{\alpha\beta}(\mathbf{k},\omega) = \sum_i \left[\left(\mathsf{T}^{+}\mathbf{V}^{\alpha+}\right) \otimes \left(\mathbf{V}^{\beta}\mathsf{T}\right)\right]_{ii} \delta(\omega - \mathsf{g}_{ii}\omega_i') \left\{ n(\omega) + \frac{1}{2}(1 - \mathsf{g}_{ii}) \right\}, \tag{21}$$

where g_{ii} is the ith diagonal element of the commutator matrix of $\mathbf{x}$.

The x-ray cross section can be directly calculated as

$$\begin{aligned} I(\mathbf{k},\omega) =& I_0 \mathbf{k}^{\intercal} \mathsf{S}_{ph}(\mathbf{k},\omega)\mathbf{k} \\ =& I_0 \sum_{\alpha} |\mathbf{V}^{\alpha}\mathsf{T}k_{\alpha}|^2 \delta(\omega - \mathsf{g}_{ii}\omega_i') \left\{ n(\omega) + \frac{1}{2}(1 - \mathsf{g}_{ii}) \right\}, \end{aligned} \tag{22}$$

where we used $\mathbf{V}_i'^{\alpha}$ instead of $\mathbf{V}_i^{\alpha}$ defined as:

$$\mathbf{V}_i'^{\alpha} = \mathbf{V}_i^{\alpha} f_i e^{-W_i(\mathbf{k})}, \tag{23}$$

with f_i being the atomic form factor and $W_i(\mathbf{k})$ the Debye-Waller factor of site i.

[1] Kim, J. & Han, J. Coupling of phonons and spin waves in a triangular antiferromagnet. *Phys. Rev. B* **76**, 054431 (2007).

[2] Toth, S. & Lake, B. Linear spin wave theory for single-Q incommensurate magnetic structures. *J. Phys. Condens. Matter* **27**, 166002 (2015).

[3] Jia, C., Nam, J. H., Kim, J. S. & Han, J. H. Lattice-coupled antiferromagnet on frustrated lattices. *Phys. Rev. B* **71**, 212406 (2005).
